# Brain swelling is independent of peripheral plasma cytokine levels in Malawian children with cerebral malaria

**DOI:** 10.1186/s12936-018-2590-0

**Published:** 2018-11-26

**Authors:** Visopo Harawa, Madi Njie, Anne Kessler, Augustine Choko, Benjamin Kumwenda, Sam Kampondeni, Michael Potchen, Kami Kim, Anthony Jaworowski, Terrie Taylor, Wilson Mandala, Karl Seydel, Stephen Rogerson

**Affiliations:** 1 Biomedical Sciences Department, College of Medicine, University of Malawi, Blantyre, Malawi; 2 Malawi-Liverpool Wellcome Trust Clinical Programme, Blantyre, Malawi; 3 Department of Medicine at the Doherty Institute, University of Melbourne, Melbourne, Australia; 4 Albert Einstein College of Medicine, Bronx, NY USA; 5 Blantyre Malaria Project, University of Malawi College of Medicine, Blantyre, Malawi; 6 University of Rochester, Rochester, NY USA; 7 London School of Hygiene & Tropical Medicine, London, UK; 8 University of South Florida, Tampa, FL USA; 9 Life Sciences Program, Burnet Institute, Melbourne, Australia; 10 Health and Biomedical Sciences, RMIT University, Melbourne, Australia; 11 Department of Osteopathic Medical Specialties, College of Osteopathic Medicine, Michigan State University, E. Lansing, MI USA; 12 Academy of Medical Sciences, Malawi University of Science and Technology, Thyolo, Malawi

**Keywords:** Cerebral malaria, Brain swelling, Cytokines, *Plasmodium falciparum*, Africa

## Abstract

**Background:**

Cerebral malaria (CM) is often fatal, and severe brain swelling is a predictor of CM-related mortality. CM is characterized by elevated circulating pro-inflammatory cytokines TNF and IFN-γ and anti-inflammatory cytokine IL-10, however whether cytokine levels correlate with brain swelling severity is unknown. This study therefore was conducted to investigate the relationship between cytokine levels and brain swelling severity in children presenting with CM.

**Methods:**

A total of 195 Malawian children presenting with CM were recruited and had the concentrations of plasma cytokines determined and compared to brain swelling severity, determined by MRI examination, and graded as severe, moderate, mild or none.

**Results:**

Levels of IL-1β, IL-6, IL-8 and IL-10 did not differ between CM patients with and without severe brain swelling. Compared to children without brain swelling, IL-12 levels were higher in children with severe swelling (p < 0.01, no swelling 1 pg/mL, IQR [1] vs. severe swelling 18.7 pg/mL, IQR [1–27]), whereas TNF concentrations were higher in children with moderate brain swelling compared to children with no swelling (p < 0.01, no swelling 3 pg/mL, IQR [1–20] vs. moderate swelling 24 pg/mL, IQR [8–58]. Multivariate analysis showed that no single cytokine independently predicted brain swelling.

**Conclusion:**

Severe brain swelling in paediatric CM was independent of tested blood pro-inflammatory and anti-inflammatory cytokines which are markers of systemic inflammation.

**Electronic supplementary material:**

The online version of this article (10.1186/s12936-018-2590-0) contains supplementary material, which is available to authorized users.

## Background

Despite substantial progress in reducing the burden of malaria globally [1], *Plasmodium falciparum* malaria still accounts for hundreds of thousands of paediatric deaths annually in sub-Saharan Africa [2]. The exact mechanisms that contribute to death are not fully known. Clinically, *P. falciparum* malaria infections present with a spectrum of severity ranging from asymptomatic infections to uncomplicated malaria illness (UM), and more severe forms of disease including cerebral malaria (CM), severe malarial anaemia and/or respiratory distress [3]. CM is the major contributor to mortality [3] and often results in debilitating neurological impairments in survivors [4]. Although some investigators have reported an association between overproduction of some pro-inflammatory cytokines, such as tumour necrosis factor (TNF), interferon gamma (IFN-γ), interleukin 6 (IL-6) and interleukin 1 beta (IL-1β) and CM pathogenesis [5], disease severity and death [6] others reported lack of association between TNF concentration and malaria severity in Ugandan children [7].

Studies have shown that inflammatory cytokines can affect the integrity and functions of the blood brain barrier (BBB) leading to vasogenic oedema and protein extravasation [8–10]. Previous work has identified increased intracranial pressure and brain swelling in paediatric patients with CM [4, 11], and recent work conducted in Malawi identified brain swelling, as shown by magnetic resonance imaging (MRI), as a key risk factor for a fatal outcome [12]. The mechanisms and processes leading to brain swelling in CM remain uncharacterized. Here, the association was investigated between severity of brain swelling on MRI and peripheral blood cytokine levels in children with clinical CM to expand on the current understanding of the role of inflammatory processes in CM pathogenesis. Malaria retinopathy was included in the definition of CM to improve specificity. In addition, the relationships between cytokine concentrations and two clinical parameters: duration of coma and parasite density were investigated. The two clinical parameters were investigated because total parasite load measured as plasma HRP-2 concentration has previously been shown to predict progression to CM [13], and pro-inflammatory cytokines TNF and IFN-γ and anti-inflammatory cytokine interleukin 10 (IL-10) have been associated with CM [5, 14, 15].

## Methods

### Study area and study population

Children aged between 6 months and 12 years old presenting with CM at Queen Elizabeth Central Hospital (QECH) in Blantyre, Malawi were recruited from January 2009 to June 2016. CM was defined based on the WHO definition of fever (temperature > 37.5 °C) with asexual stage *P. falciparum* parasites on blood film microscopy combined with a Blantyre coma score of 2 or less at admission and 4 h later, after eliminating other potential causes of seizures, such as hypoglycaemia. Coma duration prior to presentation was determined by asking the guardian the time the child became comatose.

All study participants underwent a direct and indirect dilated ophthalmological funduscopic examination. Children with retinal findings characteristic of malaria, including retinal whitening, haemorrhages and vessel discoloration [16, 17], were classified as retinopathy positive (Ret+ CM) and children with normal ocular fundi as retinopathy negative (Ret− CM). The WHO criteria for CM are highly sensitive for true CM, but the inclusion of retinal examination has been shown to improve CM specificity. Parasitaemic children with Ret− CM are commonly found to have a non-malaria aetiology of coma [12, 17]. Therefore, only Ret+ CM cases were included in the analysis. Data from HIV-infected participants were excluded from the final analysis since studies done in this population have shown that that there is synergy between the two infections [9], and that HIV infection on its own also has an independent effect on the cytokine profiles of the infected individuals [18]. Informed consent was obtained from parents or guardians for all the children enrolled in the study. A 5 mL venous blood sample was collected in sodium heparin tubes from each participant at recruitment. After centrifugation, plasma was stored at − 80 °C until the day of analysis. Participants from 2009 to 2013 were treated with IV quinine, whereas patients from 2014 to 2016 were treated with IV artesunate as recommended by the National Malaria Control Programme of Malawi.

### Brain swelling analysis

Brain scans were performed using a 0.35-T Signa Ovation Excite MRI scanner (General Electric, Milwaukee, USA). Two radiologists, unaware of each other’s readings and each patient’s retinopathy status and clinical outcome, interpreted each MRI scan. Brain swelling was graded for severity on the basis of pre-specified criteria [12]. Overall brain swelling was scored on the basis of the appearance of the cerebral hemispheres on a scale from 1 to 8, with a score of 1 indicating marked atrophy, 2 mild atrophy, 3 normal brain size, 4 slight swelling, 5 mild swelling, 6 moderate swelling, 7 substantial swelling with diffuse sulcal and cisternal effacement but no evidence of herniation, and 8 sulcal and cisternal effacement with evidence of herniation [12]. Scores of 7 and 8 were pre-specified as severe brain swelling because the radiologists considered these scores to indicate a life-threatening condition [12]. In this study, brain swelling was defined as follows: score of ≤ 3: normal brain size; scores of 4–5: mild swelling; score of 6: moderate swelling; scores of 7–8: severe swelling.

### Cytokine analysis

Plasma cytokine concentrations were measured using a human inflammatory cytokines cytometric bead array (CBA) (Becton–Dickinson), a multiplex assay that allows for the simultaneous quantification of IL-1β, IL-6, interleukin 8 (IL-8), IL-10, interleukin 12 (IL-12) and TNF. This combination of cytokines was chosen based on their previously reported roles in malaria and specifically in CM [15, 19]. A 50 μL aliquot of each sample was mixed with 50 μL of the capture beads mixture. Samples were diluted 1:10 using assay diluent. Subsequent steps were performed according to manufacturer’s instructions (BD CBA Instruction Manuals, 2016). Samples were acquired on CyAn ADP flow cytometer (Beckman Coulter) and analysed using BD FCAP software version 3.0 (San Jose, CA, USA).

### Statistical analysis

**Fig. 1:**
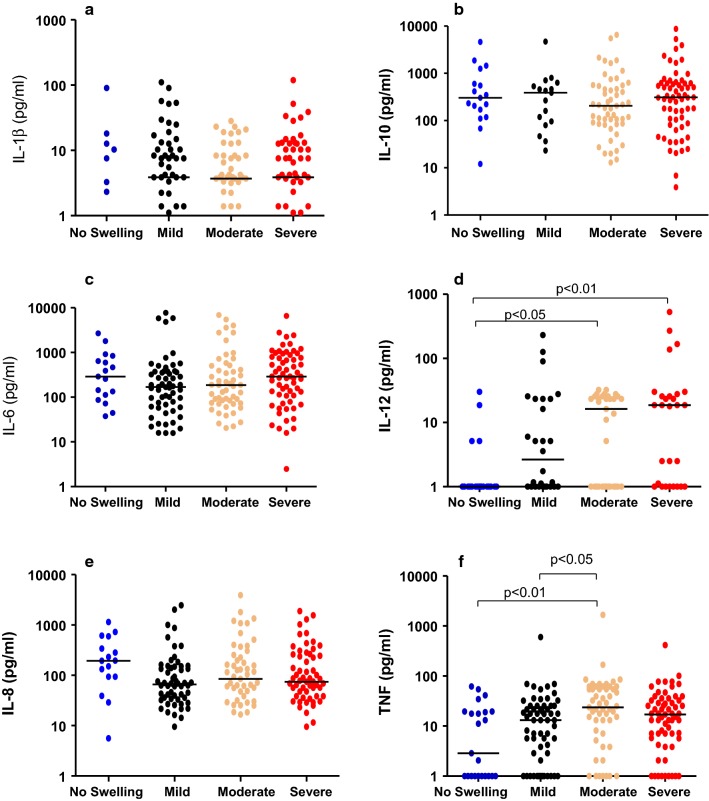
Plasma cytokine levels in CM children stratified by severity of brain swelling. Median plasma cytokine concentrations (pg/mL) at hospital presentation in CM children with varying severity of brain swelling on MRI, normal (score < 3, n = 23), mild (score 4,5, n = 58), moderate (score 6, n = 53), severe (score 7,8, n = 61); **a** IL-1β, **b** IL-10, **c** IL-6, **d** IL-12, **e** IL-8, **f** TNF. Kruskal–Wallis test was used for group analysis, p value of ≤ 0.05 was considered statistically significant. The bar represents a median value

**Fig. 2:**
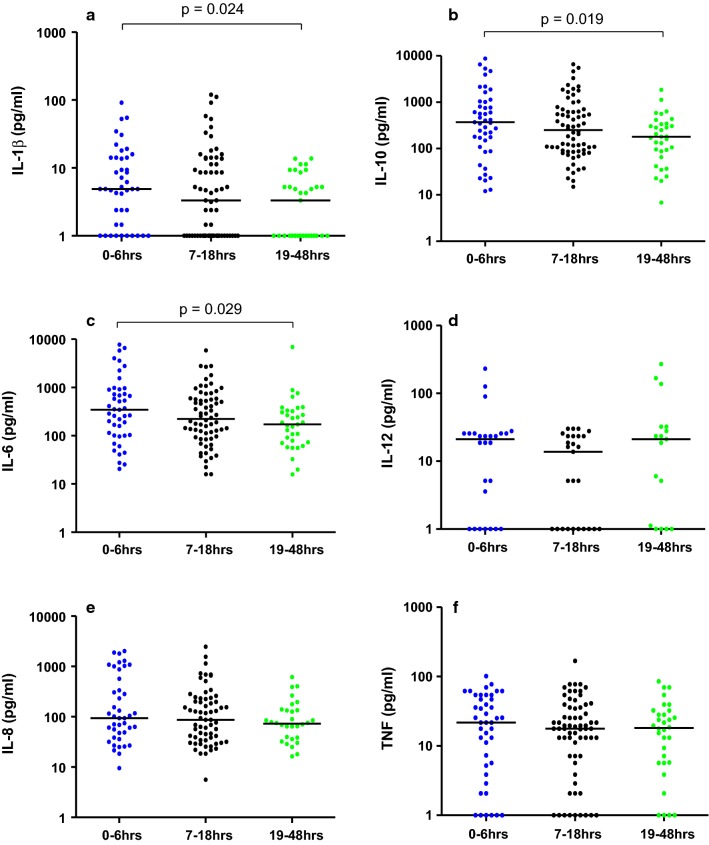
Plasma cytokine levels in CM children stratified by duration of coma prior to admission. Median plasma cytokine levels (pg/mL) at hospital presentation in CM children categorized into three groups based on the length of coma prior to admission [0–6 h (n = 45); 7–18 h, (n = 68); 19–48 h, (n = 33)]; **a** IL-1β, **b** IL-10, **c** IL-6, **d** IL-12, **e** IL-8, **f** TNF. Kruskal–Wallis test was used for paired group analysis, p value of ≤ 0.05 was considered statistically significant. The bar represents a median value. All the medians were similar for all the cytokines

**Fig. 3:**
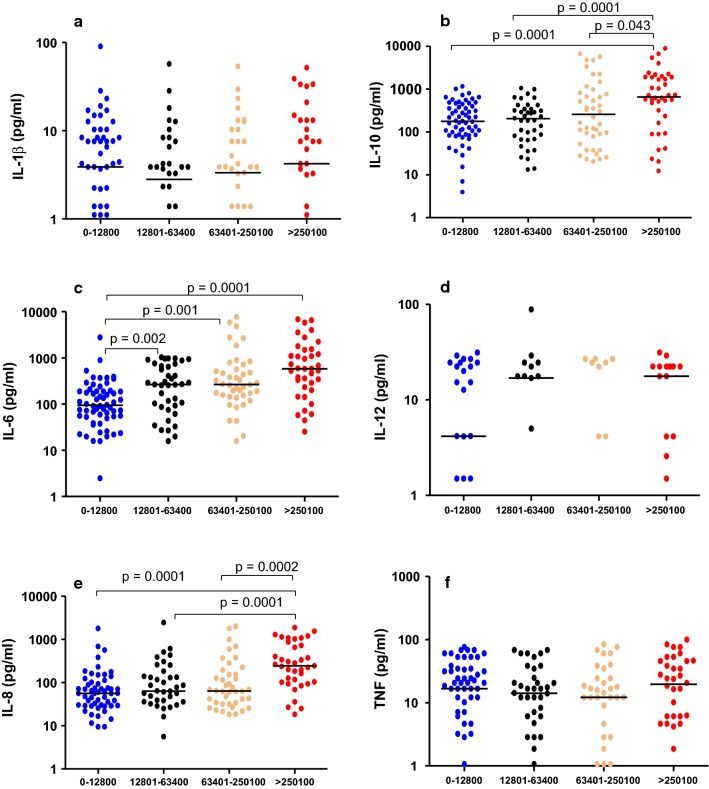
Plasma cytokine levels in CM children stratified by peripheral parasitaemia. Median plasma cytokine levels (pg/mL) at hospital presentation in children with CM, categorized into four groups based on peripheral blood parasite load per microliter of blood [1–12,800, (n = 60); 12,801–63,400, (n = 39); 63,401–250,100, (n = 42) and > 250,100, (n = 38)] at presentation; **a** IL-1β, **b** IL-10, **c** IL-6, **d** IL-12, **e** IL-8, **f** TNF. Kruskal–Wallis test was used for group analysis, p value of ≤ 0.05 was considered statistically significant. The bar represents a median value

**Fig. 4:**
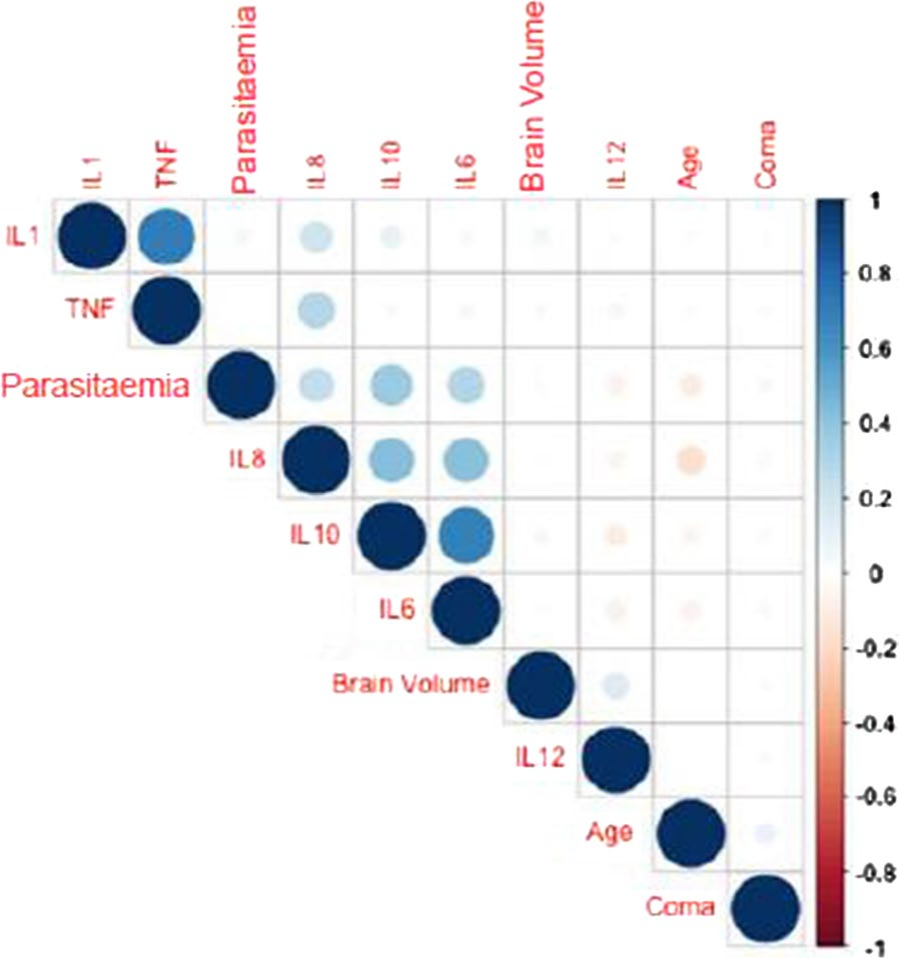
Correlation between plasma concentration of cytokines with disease parameters in children with CM. A heatmap representing the correlation between different cytokines with other variables associated with acute CM (n = 195) such as parasitaemia levels, age, length of coma and brain swelling in Malawian children. Circle size and darkness signify increased positive correlation (shade of colour is proportional to the probability of dependence between variables from + 1 to − 1)

Data were analysed using Stata version 14.0 (Stata Corp, TX, USA) and GraphPad Prism 5 (GraphPad, CA, USA). Medians and inter quartile range (IQR) were computed for continuous variables after log transformation. Kruskal–Wallis test was used to compare medians across more than two groups and between-group comparisons of cytokine concentrations for the different groups were assessed with Dunn’s multiple comparison test. Mann–Whitney U test was used to assess bivariate association with brain swelling for continuous variables that were not normally distributed. Fisher’s exact test was used to assess bivariate association with brain swelling for categorical variables because the sample size was small. Univariate and multivariate logistic regression models were fitted to investigate independent factors associated with brain swelling. The models were used to estimate the crude, adjusted odds ratios (ORs) and associated 95% confidence intervals (CI). Variables with p-value ≤ 0.2 in univariate analysis were included in the multivariate model shown in Tables 1, 2. Correlations between different variables were determined by Microsoft Excel and cytokine network analysis and a correlation matrix heatmap was done using R package version 3.2.0 presented in Fig. 4. Graphs in Figs. 1, 2, 3 were plotted using GraphPad Prism 5. Differences were considered to be statistically significant if the p values were less than or equal to 0.05.

**Table 1:** Demographic characteristics of the study participants: univariate and multivariable logistic regression model showing the association between brain swelling and demographic characteristics of the study participants

Variable	Characteristic	No severe swelling	Severe swelling	p-value*	Unadjusted			Adjusted		
					OR	95% CI	p-value**	OR	95% CI	p-value***
Gender	Female (%)	33 (37.5)	55 (62.5)	0.24	0.68	0.38; 1.22	0.195	1.04	0.28; 3.87	0.954
	Male (%)	50 (46.73)	57 (53.27)							
Age (months)	Median (IQR)	48 (31; 68)	42 (28.0; 65.5)	0.29	1	0.99; 1.00	0.523		ND	
Hours in coma prior to admission	Median (IQR)	2.08 (1.79; 2.48)	2.20 (1.79; 3.04)	0.22	1.25	0.89; 1.75	0.191	1.31	0.64; 2.66	0.462
Parasitaemia (parasites log_10_/μL)	Median (IQR)	10.83 (8.16; 11.78)	10.64 (7.46; 12.41)	0.66	1.03	0.93; 1.14	0.597	ND	ND	

In this analysis brain swelling scores of 3 to 6 were considered to not have severe swelling and scores of 7 and 8 were considered to have severe brain swelling. Variables with p-value ≤ 0.2 in univariate analysis were included in the multivariate model. Group comparisons were done using Mann–Whitney U test except for age, where Fisher’s Exact test was used. Results for parasitaemia were log transformed (log_10_)

*ND* not done

* Univariate association

** Simple logistic regression

*** Multivariable logistic regression

**Table 2:** Univariate and multivariate associations with brain swelling: univariate association and multivariable logistic regression model showing the association between brain swelling and a number of cytokines

Variable	Characteristic	No severe swelling	Severe swelling	p-value*	Unadjusted			Adjusted		
		n (%)	n (%)		OR	95% CI	p-value**	OR	95% CI	p-value***
IL-10 (pg/mL)	Median (IQR)	5.28 (4.44; 6.32)	5.42 (4.40; 6.37)	0.61	1.04	0.87; 1.26	0.644		ND	
IL-1β (pg/mL)	Median (IQR)	2.07 (1.35; 2.77)	2.02 (1.31; 2.58)	0.69	1	0.79; 1.27	0.996		ND	
IL-6 (pg/mL)	Median (IQR)	5.11 (4.23; 5.95)	5.27 (4.29; 6.38)	0.26	1.11	0.91; 1.35	0.297		ND	
IL-12 (pg/mL)	Median (IQR)	2.73 (1.42; 3.20)	3.11 (2.87; 3.29)	0.05	1.37	0.94; 1.99	0.103	1.24	0.74; 2.08	0.415
IL-8 (pg/mL)	Median (IQR)	4.27 (3.58; 5.04)	4.46 (3.65; 5.50)	0.16	1.19	0.93; 1.52	0.159	1.91	0.98; 3.71	0.056
TNF (pg/mL)	Median (IQR)	2.82 (2.26; 3.32)	3.03 (2.50; 3.82)	0.07	1.24	0.95; 1.63	0.119	1.41	0.76; 2.76	0.275
Ratio (IL10/TNF)	Median (IQR)	2.24 (1.32; 3.45)	2.23 (0.85; 3.58)	0.77	0.96	0.82; 1.13	0.662		ND	
Ratio (IL-10/IL-6)	Median (IQR)	0.20 (0.51; 0.85)	0.14 (0.73; 0.77)	0.44	0.9	0.69; 1.18	0.448		ND	
Ratio (IL-10/IL-1β)	Median (IQR)	3.43 (2.26; 4.51)	3.62 (2.37; 4.46)	0.96	1	0.83; 1.21	0.962		ND	
Ratio (IL-10/IL-12)	Median (IQR)	2.57 (1.07; 3.92)	2.64 (0.68; 3.55)	0.69	0.94	0.75; 1.19	0.632		ND	
Ratio (IL-10/IL-8)	Median (IQR)	0.96 (0.12; 1.72)	0.92 (0.33; 1.72)	0.67	0.93	0.76; 1.14	0.496		ND	

In this analysis brain swelling scores of 3 to 6 were considered to not have severe swelling and scores of 7 and 8 were considered to have severe swelling. Variables with p-value ≤ 0.2 in univariate analysis were included in the multivariate model. Group comparisons were done using Mann–Whitney U test. Results for cytokine concentrations and parasitaemia were log transformed (log_10_). Multivariable logistic regression model analysis was based on only those variables with p-value ≤ 0.2 in univariate analysis

*ND* not done

* Univariate association

** Simple logistic regression

*** Multivariable logistic regression

## Results

### Demographic characteristics of study patients

A total of 195 HIV-uninfected children with CM and malarial retinopathy aged between 6 months and 12 years were studied. Convenience sampling was used based on the availability of plasma samples and MRI data. Sample collection dates span from 2009 to 2015. Of the 195 children, 107 (55%) were male. Twenty-eight (14%) study participants died. Based on the MRI interpretation [20], 22 children had no brain swelling, 58 had mild swelling, 53 had moderate swelling and 62 had severe brain swelling. Previous published work demonstrated a correlation between severe swelling and death [12]; hence during analysis the children with no severe swelling were compared with those that had severe swelling (Tables 1, 2).

### Univariate and multivariate associations of cytokines with brain swelling

In univariate analyses there were no differences in cytokine levels between children with no severe swelling and those with severe swelling (Table 2). Variables with p ≤ 0.2 in univariate analysis (IL-8, IL-12 and ratio TNF) were included in the multivariate model. Just as in univariate analysis, there were no independent predictors of brain swelling with variables included in the model (Table 2). There were also no differences in cytokine levels as well as demographic characteristics between children with no swelling and those with swelling (Additional file 1: Tables S1, S2, respectively).

### Plasma cytokine levels in children with CM and different degrees of brain swelling

Plasma levels of the following cytokines did not significantly differ between children with severe brain swelling and those who had no swelling, mild or moderate swelling (Fig. 1a–f): IL-1β, TNF, IL-10, IL-6, and IL-8. Children with severe brain swelling had significantly higher levels of IL-12 than those with no swelling: 18.7 pg/mL [1–27] vs. 1 pg/mL [1] (p = 0.01) (Fig. 1d). Children with moderate brain swelling had significantly higher TNF levels compared to those who had no swelling: 24 pg/mL [8–58] vs. 3 [1–20] (p < 0.01), and those who had mild swelling: 13 pg/mL [3–25] (p < 0.05) (Fig. 1f).

### IL-1β, IL-6, IL-10 plasma levels were lower in those with longer coma duration prior to admission

Because many cytokines have short half-lives in plasma spanning 4 to 12 h [18], the relationship between plasma cytokine levels and the estimated time the children were in coma before presenting at the hospital for admission was investigated. CM pathogenesis is likely a progressive process. It was therefore hypothesized that the time the disease remains untreated (i.e., the time from initial onset of coma to presentation at the Paediatric Research Ward) is an important determinant of disease severity. Although time in coma prior to presentation to the hospital is only an approximation of the onset of the disease process, it is used as an indicator of progression along this disease pathway. Given the transient kinetics of peripheral cytokines, consideration of the time from initial disease onset might be important.

Based on the distribution of the coma duration prior to admission, children were stratified into three groups: 0–6 h coma, 7–18 h, 19–48 h. Compared to children who presented with ≥ 19 h after coma onset, children who presented with ≤ 6 h of coma prior to admission had significantly higher levels of the cytokines IL-1β: 5 pg/mL [1.46–14] vs. 3 [1–7], (p = 0.024) (Fig. 2a), IL-10: 369 pg/mL [129–1030] vs. 180 [77–340], (p = 0.019), (Fig. 2b), and IL-6: 345 pg/mL [102–907] vs. 172 [72–327], (p = 0.029) (Fig. 2c).

### Plasma cytokine levels of IL-6, IL-8 and IL-10 were associated with high parasitaemia

The relationship between peripheral cytokine levels and parasite density on admission was investigated. Study participants were stratified into four groups based on quintile ranges of parasite density per microlitre of blood (1–12,800, 12,801–63,400, 63,401–250,100, and > 250,100). Plasma cytokine levels of IL-10: (657 [212–1868] vs. 177 [85–423] pg/mL) (Fig. 3b); IL-6: (584 [225–1512] vs. 94 [53–190] pg/ml) (Fig. 3c), and IL-8: (245 [104–802] vs. 57 [29–95] pg/mL) (Fig. 3e) were significantly higher in children in the highest parasitaemia quintile (> 250,100) compared to those with the lowest parasite density (1–12,800) (p < 0.0001). Concentrations of the cytokines IL-10, IL-8 and IL-6 correlated with parasite density in the correlation analysis (Fig. 4).

### Cytokine network and correlation with brain swelling in cerebral malaria

To investigate the relationships between peripheral blood concentrations of the cytokines IL-10, IL-1β, IL-6, IL-8, IL-12, and TNF and age, parasitaemia, length of coma, and brain swelling, correlation analyses were performed. There were correlations between levels of some of the cytokines: IL-8 correlated with IL-1β, IL-6, and TNF, whereas IL-10 correlated with IL-8 and IL-6. This result demonstrates that cytokines work in concert and provide a plasma cytokine profile in CM in children. This cytokine network is critical in driving the pro-inflammatory response as well as immune response counter regulation. As shown in Fig. 4, there was no association between cytokine concentrations and brain swelling or duration of coma.

## Discussion

Cerebral malaria has a high mortality rate but an understanding of the mechanisms leading to death remains unclear. Factors including inflammatory cytokines [5, 15], markers of endothelial activation [21, 22], coagulation dysfunction [23], and total parasite load [24] have all been implicated, and combinations of these biomarkers can improve predictive value [25]. More recently, a strong association was described between severity of brain swelling on MRI and mortality from CM in Malawian children, with 84% of children who died having severe brain swelling [12]. Given the strong associations between elevations of certain systemic cytokines, disease severity [5, 15] and outcome [26] it was hypothesized that an elevated systemic inflammatory response, as indicated by elevated plasma cytokine concentrations of inflammatory cytokines IL-1β, TNF, IL-6, and IL-8, may be directly or indirectly associated with brain swelling. In a unique series of 195 children with CM who underwent MRI scanning, concentrations of a selection of pro- and anti-inflammatory cytokines were measured in blood plasma collected at hospital presentation, and the relationships were investigated between plasma cytokines, brain swelling, coma duration, and parasite density in these children.

Overall, there were weak associations between pro- and anti-inflammatory cytokine concentrations and brain swelling, possibly reflecting the different temporal dynamics of cytokines and brain swelling, or the difficulties of assessing tissue-specific inflammation in peripheral blood. Higher concentrations of IL-12 in patients with severe and moderate brain swelling compared to patients without swelling, and of TNF in moderate (but not severe swelling) compared to children without swelling were found, but concentrations of other cytokines did not differ with the severity of the brain swelling. Data on the relationship between IL-12 and CM are limited [27]. In contrast, studies aimed at investigating the association between high TNF levels and malaria severity as well as death have yielded conflicting results with some studies showing an association [5, 28, 29] and another study showing no association [7]. The weak relationship between circulating TNF and brain swelling observed may be because local production of TNF at sites of sequestration is critical, and is not accurately reflected in circulating levels. Additionally, the lack of association between severe brain swelling and TNF concentrations may reflect differences in the kinetics of the different processes, with cytokine concentrations potentially peaking earlier in the disease process than the brain swelling.

To investigate how differences in duration of coma might affect cytokine concentrations, children were categorized according to duration of coma prior to admission. Children who presented within 6 h of coma onset had higher concentrations of IL-1β, IL-6, and IL-10 than those who presented more than 18 h after coma onset. While the precise time of onset of the malaria episode was not known, loss of consciousness implies significant neurological dysfunction. Coma duration was not associated with CM brain swelling. It is possible that the onset of coma is associated with some kind of a cytokine storm that gradually diminishes as cytokine levels decline with time [30, 31]. This suggests different kinetics to the two processes.

Parasite biomass, measured as concentration of plasma HRP2 (a biomarker of sequestered and circulating parasites) has previously been correlated with malaria severity and progression to CM [13, 32], and it is this sequestration that is thought to directly or indirectly contribute to CM pathogenesis through activation of the inflammasome pathway in endothelial cells [33] and possibly by inducing endothelial barrier disruption [34]. Some researchers have demonstrated a strong association between coma, congestion and microvascular obstruction in human CM leading to the hypothesis that CM brain swelling could, in part, be caused by congestion and increased hydrostatic pressure which leads to oedema [24]. Although there were strong associations between peripheral blood parasite density and concentrations of cytokines IL-6, IL-8 and IL-10, there was no significant association between peripheral blood parasite density and severe brain swelling (Fig. 4). The observed association between peripheral parasite density and cytokine levels may indicate compartmentalized immune response to circulating parasitaemia [35].

Cytokines, such as TNF, IFN-γ, IL-6 and IL-1β, are likely to be beneficial in the early stages of the infection in parasite clearance, but might actually contribute to the pathogenesis associated with CM [36], and to disease severity and death [37] if produced in an unregulated manner. In contrast, anti-inflammatory cytokines, including IL-10, are also elevated in children with CM, but are thought to be involved in counteracting the effect of the pro-inflammatory cytokines and down-regulating their production [38]. The findings of this study are instead more consistent with inflammatory and counter-regulatory cytokine responses that are appropriately increased as parasite burden increased. In a heat map analysis, there were no significant correlations between cytokine concentrations and brain swelling (Fig. 4), suggesting that peripheral blood cytokine concentrations were not major drivers of brain swelling in this population. Overall, the results of this and previous studies concerning cytokines in malaria suggest that peripheral cytokine levels are elevated in CM but that within the CM cases circulating cytokines appear not to correlate with either brain swelling or death [5]. If cytokines do not directly contribute to brain swelling, there must be other signals that initiate and/or perpetuate swelling (such as an increased responsiveness of the endothelial cells to cytokines or cytokine-independent activation of the endothelial cells).

The strengths of this study include the large number of children undergoing an MRI scan, and the use of retinal examination, resulting in stringently defined CM [17]. By analysing plasma cytokine levels in 195 children with CM, the study had sufficient statistical power (> 80%) to observe significant differences in the various parameters tested. Limitations include only a single time point for blood sampling, which varied between children relative to onset of coma and probably to time of infection. Blood samples collected at different time points from onset of coma for each child would have permitted exploration of the relationship between cytokine levels and duration in coma in more detail. Second, like all human cohort studies of pre-fatal CM, samples from this study were collected from the peripheral blood, and not from the sites of parasite sequestration and disease pathology. Peripheral blood cytokine concentrations may not accurately reflect those at the site of disease in the cerebral vasculature. There is evidence that inflammatory cytokines are produced within the central nervous system [39], and a recent study reported higher cytokine concentrations in cerebral spinal fluid (CSF) than in plasma from CM patients [39], although a second study did not find any correlation between CSF and serum cytokine levels [40]. Whether measurement of cytokines in CSF, or in brain tissue, would provide stronger correlates of brain swelling, remains to be studied.

## Conclusions

Although inflammatory cytokine concentrations in peripheral blood at time of presentation are associated with parasite load and time of coma onset, they do not appear to directly correlate with brain swelling severity on MRI in strictly defined CM. These findings might reflect the different temporal dynamics of cytokines and brain swelling, or the difficulties of assessing tissue-specific inflammation in peripheral blood. Further investigation should study the relationship between markers of endothelial cell activation and brain swelling.

## Additional file

**Additional file 1: Table S1.** Demographic characteristics of the study participants. **Table S2.** Univariate and multivariate associations with brain swelling.

## Abbreviations

BBB: blood brain barrier
CBA: cytometric bead array
CI: confidence interval
CM: cerebral malaria
COMREC: College of Medicine Research and Ethics Committee
HIV: human immunodeficiency virus
HRP2: histidine-rich protein 2
IFN-γ: interferon gamma
IL: interleukin
iRBCs: infected red blood cells
IV: intravenous
MRI: magnetic resonance imaging
MSU-IRB: Michigan State University Institutional Review Board
OR: odds ratio
QECH: Queen Elizabeth Central Hospital
Ret−: retinopathy negative
Ret+: retinopathy positive
TNF: tumour necrosis factor
UM: uncomplicated malaria
WHO: World Health Organization
