# Supplementary material for: Brain swelling is independent of peripheral plasma cytokine levels in Malawian children with cerebral malaria
Source: Malar J. 2018 Nov 26;17:435. doi: 10.1186/s12936-018-2590-0 (PMC6260579; doi:10.1186/s12936-018-2590-0)
Supplement: Supplementary file 1 — Additional file 1: Table S1. Demographic characteristics of the study participants. Table S2. Univariate and multivariate associations with brain swelling. [file 12936_2018_2590_MOESM1_ESM.docx]

**Table S1:** Demographic characteristics of the study participants

|  |  |  |  |  |  |  |  |  |  |  |  |
| --- | --- | --- | --- | --- | --- | --- | --- | --- | --- | --- | --- |
|  |  |  |  |  |  | **Crude** |  |  | **Adjusted** |  |  |
|  | **Variable** | **Characteristic** | **No brain swelling** | **Brain swelling** | **p-value*** | **OR** | **95% CI** | **p-value**** | **OR** | **95% CI** | **p-value***** |
|  | Sex | Female (%) | 12 (13.6) | 76 (86.4) | 0.371 |  |  |  |  |  |  |
|  |  | Male (%) | 10 (9.4) | 97 (90.6) |  | 1.53 | 0.63; 3.73 | 0.349 | ND |  |  |
|  | Age (months) | Median (IQR) | 36.5 (22.0; 58.0) | 46.0 (31.0; 67.0) | 0.114 | 1.02 | 1.0; 1.04 | 0.107 | 1.01 | 0.99; 1.03 | 0.548 |
|  | Hours in coma prior to admission | Median (IQR) | 2.19 (1.95; 2.40) | 2.08 (1.79; 2.99) | 0.78 | 1.06 | 0.64; 1.76 | 0.811 | ND |  |  |
|  | Parasitaemia (parasites log10/uL) | Median (IQR) | 11.34 (10.07; 12.55) | 10.64 (7.46; 12.15) | 0.32 | 0.93 | 0.78; 1.12 | 0.454 | ND |  |  |
|  |  |  |  |  |  |  |  |  |  |  |  |
|  |  |  |  |  |  |  |  |  |  |  |  |
|  | * Univariate association  **Simple Logistic regression | |  |  |  |  |  |  |  |  |  |
|  | ***Multivariable logistic regression | |  |  |  |  |  |  |  |  |  |
|  | ND Not done |  |  |  |  |  |  |  |  |  |  |
|  |  |  |  |  |  |  |  |  |  |  |  |

Univariate and multivariable logistic regression model showing the association between brain swelling and demographic characteristics of the study participants. In this analysis brain swelling score of 3 was considered to not have swelling and scores of 4-8 were considered to have brain swelling. Variables with p-value ≤ 0.2 in univariate analysis were included in the multivariate model. Group comparisons were done using *Mann-Whitney U* test except for age where Fisher’s Exact test was used. Results for parasitaemia were log transformed (log_10_).

|  |  | |  | |  |  |  |  |  |  |  |  |
| --- | --- | --- | --- | --- | --- | --- | --- | --- | --- | --- | --- | --- |
| **Table S2:** Univariate and multivariate associations with brain swelling | | | | | |  |  |  |  |  |  |  |
|  |  | |  | |  |  | **Crude** |  |  | **Adjusted** |  |  |
| **Variable** | **Characteristic** | | **No brain swelling n (%)** | | **Brain swelling n (%)** | **p-value*** | **OR** | **95% CI** | **p-value**** | **OR** | **95% CI** | **p-value***** |
| IL-10 (pg/mL) | Median (IQR) | | 5.4 (2.86; 6.39) | | 5.36 (4.44; 6.34) | 0.67 | 1.11 | 0.83; 1.48 | 0.468 | ND |  |  |
| IL-1β (pg/mL) | Median (IQR) | | 2.33 (1.88; 2.90) | | 2.02 (1.36; 2.70) | 0.62 | 0.9 | 0.55; 1.46 | 0.669 | ND |  |  |
| IL-6 (pg/mL) | Median (IQR) | | 5.54 (4.28; 6.39) | | 5.18 (4.28; 6.22) | 0.73 | 0.97 | 0.72; 1.31 | 0.844 | ND |  |  |
| IL-12 (pg/mL) | Median (IQR) | | 2.94 (1.42; 3.20) | | 3.11 (2.43; 3.29) | 0.56 | 1.09 | 0.64; 1.88 | 0.748 | ND |  |  |
| IL-8 (pg/mL) | Median (IQR) | | 5.02 (3.83; 5.64) | | 4.32 (3.58; 5.22) | 0.16 | 0.82 | 0.57; 1.18 | 0.289 | ND |  |  |
| TNF (pg/mL) | Median (IQR) | | 2.81 (2.32; 3.51) | | 3.03 (2.41; 3.65) | 0.59 | 1.12 | 0.74; 1.70 | 0.58 | ND |  |  |
| Ratio (IL10/TNF) | Median (IQR) | | 2.27 (0.32; 4.32) | | 2.21 (1.0; 3.58) | 0.77 | 1.02 | 0.79; 1.32 | 0.86 | ND |  |  |
| Ratio (IL-10/IL-6) | Median (IQR) | | 0.29 (-0.83; 0.81) | | 0.21 (-0.64; 0.80) | 0.28 | 1.3 | 0.87; 1.93 | 0.195 | 1.23 | 0.72; 2.08 | 0.464 |
| Ratio (IL-10/IL-1β) | Median (IQR) | | 3.49 (2.55; 6.10) | | 3.52 (2.31; 4.46) | 0.55 | 0.79 | 0.50; 1.24 | 0.303 | ND |  |  |
| Ratio (IL-10/IL-12) | Median (IQR) | | 1.57 (-0.12; 3.52) | | 2.67 (0.76; 3.93) | 0.26 | 1.23 | 0.82; 1.82 | 0.313 | ND |  |  |
| Ratio (IL-10/IL-8) | Median (IQR) | | 0.27 (-0.60; 1.51) | | 1.06 (-0.06; 1.74) | 0.14 | 1.24 | 0.91; 1.70 | 0.174 | 1.14 | 0.77; 1.70 | 0.516 |
| * Univariate association  **Simple Logistic regression | | | |  |  |  |  |  |  |  |  |  |
| ***Multivariable logistic regression | | | |  |  |  |  |  |  |  |  |  |
| ND Not done | |  |  |  |  |  |  |  |  |  |  |  |

Univariate association and multivariable logistic regression model showing the association between brain swelling and a number of factors. In this analysis brain swelling scores of 3 were considered to not have swelling (normal) and scores of ≥4-8 were considered to have swelling. Variables with p-value ≤ 0.2 in univariate analysis were included in the multivariate model. Group comparisons were done using *Mann-Whitney U* test except for age where Fisher’s Exact test was used. Results for cytokine concentrations and parasitemia were log transformed (log_10_). multivariable logistic regression model. Multivariable logistic regression model analysis was based on only those variables with p-value ≤ 0.2 in univariate analysis.
